# Dental caries is associated with severe periodontitis in Chilean adults: a cross-sectional study

**DOI:** 10.1186/s12903-019-0975-2

**Published:** 2019-12-10

**Authors:** Franz-Josef Strauss, Iris Espinoza, Alexandra Stähli, Mauricio Baeza, Ricardo Cortés, Alicia Morales, Jorge Gamonal

**Affiliations:** 1 Department of Conservative Dentistry, Faculty of Dentistry, Universidad de Chile, Olivos 943, Comuna de Independencia, Santiago, Chile; 2 Department of Oral Biology, Medical University of Vienna, Vienna, Austria; 3 Center for Epidemiology and Surveillance of Oral Diseases (CESOD), Santiago, Chile; 4 Department of Periodontology, School of Dental Medicine, University of Bern, Bern, Switzerland; 5 Department of Prosthetic Dentistry, Faculty of Dentistry, Universidad de Chile, Santiago, Chile

**Keywords:** Caries, Periodontitis, Prevalence, Co-occurrence, Epidemiology, Chile

## Abstract

**Background:**

The co-occurrence of caries and periodontitis and a possible association is still a matter of debate. Thus, the aim of the study was to determine the co-occurrence of caries and periodontitis in Chilean adults.

**Methods:**

Evaluation of periodontal and dental status in 994 adults (35–44 years old) based on the First Chilean National Examination Survey 2007–2008. The prevalence of caries was defined as the percentage of participants with one or more teeth with untreated caries by using the D component of the DMFT index (DT ≠ 0). The prevalence of periodontitis was determined using standard case definitions for population-based surveillance of periodontitis described by the CDC–AAP.

**Results:**

Individuals with caries had an approximately 40% higher prevalence of severe (29.3% vs 20.8%, *p* < 0.05) and a 13% higher prevalence of total periodontitis (89.3% vs 78.4%, p < 0.05) than those without caries. Ordinal logistic regression revealed a positive association between periodontitis and the number of teeth with caries (DT ≠ 0; 3 or 4 teeth with caries: OR 1.74; CI = 1.12–2.29 *p* < 0.05; 5 or more teeth with caries: OR 2.47; CI = 1.66–3.67 *p* < 0.01).

**Conclusion:**

Dental caries is associated with the severity and prevalence of periodontitis in Chilean adults. Individuals with 3 or more teeth with untreated caries are more likely to suffer from periodontal disease.

## Background

Dental caries and periodontitis constitute a global public health problem [1, 2] and represent the main cause of tooth loss in the adult population [3, 4]. Over the last 3 to 4 decades the prevalence of caries has declined for all age groups and in many regions of the world [5] being unequally distributed among socio-economic groups [2, 6]. However, untreated caries in permanent teeth still constitute the most prevalent disease across the globe [1]. On the other hand, the prevalence of severe periodontitis has remained static in the global population and there is insufficient evidence for a declining trend in periodontitis prevalence [5]. Although caries and periodontitis are the most frequent oral diseases, surprisingly the co-occurrence of both conditions has been poorly investigated.

Available evidence on the co-occurrence of caries and periodontitis is conflicting. Consequently, a positive or a negative association between both diseases is still a matter of debate. For example, early studies have reported positive [7] and negative associations [8] but also a lack of association [9]. A recent national study in Finland showed that both conditions affect the same individuals especially in patients with severe periodontitis, suggesting a positive association [10]. It is interesting to note, however, that to date very little scientific data exists to support these findings. Indeed, and owing to the lack of evidence in this topic, the joint workshop of the European Federation of Periodontology (EFP) and the European Organization for Caries Research (ORCA) recently reported on the global burden of dental caries and periodontal diseases [11] concluding that there was a surprising paucity of robust studies analysing the co-occurrence of caries and periodontitis. Hence, there is a clear demand for further research that analyses the co-occurrence of both conditions in order to elucidate whether there is an association between caries and periodontitis.

Apart from the bacterial aetiology [12, 13], a positive co-occurrence between caries and periodontitis can be hypothesized by common risk factors such as nutrition, or poor oral hygiene. Strong evidence shows that diet has an influence on caries and emerging evidence shows an influence on periodontal diseases [2]. Furthermore, socio-behavioural aspects and the socio-economic status (SES) are also associated with the development of caries and periodontitis [14]. SES is able to influence behavioural parameters [15], such as smoking, or dental awareness to seek professional treatment. Consequently, it is plausible to suggest a positive co-occurrence of caries and periodontitis. Thus, the aim of the study was to investigate the co-occurrence of dental caries and periodontitis in Chilean adults between 35 and 44 using the data from the First Chilean National Dental Examination Survey (2007–2008).

## Methods

### Sampling and sample size

The first Chilean National Examination Survey was conducted between 2007 and 2008. The protocol had been approved by the ethical committee of the Faculty of Medicine of the University of Chile, Chile [16]. A stratified, multistage probability design was applied to divide the Chilean population in two age cohorts (young adults aged 35 to 44 and elderly adults aged 65 to 74 [16]. Study participants were recruited in 15 administrative regions. The sample size was calculated estimating a 80% prevalence of mild to severe periodontitis in Chile. In order to achieve a 95% precision rate with a range error of 0.02%, 1092 young adults and 469 senior adults were examined. The present analysis only included the younger cohort of adults between 35 and 44 years old with a complete record of periodontal parameters and caries lesions (*n* = 994). The older cohort of adults (65–74 years) were not included in the analysis due to the high prevalence of edentulism (20%) as we determined in our previous study [16]. Complete dental examinations were performed in each individual by calibrated examiners. All examiners received theoretical classes, clinical training, and calibration by a senior member of the Periodontal Department of the Faculty of Dentistry, University of Chile (JG) [16]. Calibration training was performed on successive days during which groups of 20 subjects were examined. All examinations were repeated until acceptable consistency was achieved determined by intraclass and interclass correlation coefficients. Validity and reliability examinations were performed before, during, and at the end of the study. Clinical evaluations were carried out in dental clinics from the public primary care system. The study protocol was explained to all patients, and informed consent forms were signed prior to entry in the study. To determine the impact of social, economic and environmental factors on oral health, information about the behavioural and socio-demographic characteristics were gathered through a personal interview. Individuals were classified by their educational level, which was categorised by the amount of education years in < 12 or ≥ 12 years. Household income was categorized according the national minimal monthly salary of < $286,000 or ≥ $286,000 Chilean Pesos (CLP). Individuals were classified as current smokers or non-smokers/former smokers. Diabetes mellitus was recorded as self-reported.

### Definition of variables

Prevalence of caries was determined as the percentage of those subjects with one or more teeth with caries (DT ≠ 0), obtained by the D component of the DMFT index [10]. Periodontitis was determined according to suggested standard case definitions for population-based surveillance of periodontitis described by the Centre for Disease Control and Prevention and the American Academy of Periodontology (CDC–AAP) [17, 18]. Mild periodontitis was defined as ≥2 interproximal sites with CAL ≥3 mm and ≥ 2 interproximal sites with PD ≥4 mm (not on the same tooth) or one site with PD ≥5 mm. Moderate periodontitis was defined as ≥2 interproximal sites with CAL ≥4 mm (not on the same tooth) or ≥ 2 interproximal sites with PD ≥5 mm, also not on the same tooth. Severe periodontitis was defined as having ≥2 interproximal sites with CAL ≥6 mm (not on the same tooth) and ≥ 1 interproximal sites with PD ≥5 mm. Prevalence of periodontitis was defined as the presence of mild, moderate or severe periodontitis. PD was recorded at 6 sites per tooth (mesiobuccal, buccal, distobuccal, distolingual, lingual and mesiolingual), which was assessed through a manual periodontal probe (UNC15, HuFriedy, Chicago, IL, USA) excluding the third molars. For the analysis of the co-occurrence of dental caries and periodontitis, the prevalence and severity of periodontitis among subjects with and without caries was determined.

### Statistical analysis

Categorical values are presented as percentages and continuous data are displayed as means ± SD. The data were stratified according to sociodemographic, behavioural, caries and periodontal status. Chi-square and Kruskal-Wallis tests were performed to compare caries and periodontitis according to the sociodemographic data. To determine the association between dental caries and periodontitis an ordinal logistic regression model was used. The primary outcome of the analysis was the severity of periodontitis with no-periodontitis as the reference category. In addition, age, gender, smoking status (smoker or non-smoker/former smoker), education (< 12 years or ≥ 12 years), Household income (< $286,000 or ≥ $286,000 Chilean Pesos CLP), diabetes (self-reported) and number of caries (0, 1, 2, 3–4 and ≥ 5 caries) were included as covariates in multivariate analysis. First, an analysis using uni-variate models was performed. Thereafter, a multivariate analysis model was constructed and only exposures showing in the univariable analysis associations with *p* ≤ 0.25 were included [19]. A 95% level of confidence was considered as representing statistical significance (*p* < 0.05). The statistical analysis was performed using statistical software (Stata V 12 statistical package for Mac Stata-Corp, College Station, TX, USA).

## Results

Dental records of 994 Chilean young adults were assessed. Table 1 shows the baseline characteristics of the study population. Caries prevalence was 86.0%. Participants with caries showed a higher prevalence of some form of periodontitis than individuals without caries (89.3% versus 78.4%, *p* < 0.05). Moreover, individuals with caries presented a higher prevalence of severe periodontitis than those without caries (29.3% versus 20.8%, *p* < 0.05).

**Table 1:** Characteristics of study participants with periodontal examinations according to the CDC/AAP case definition (Eke et al., 2012)

	Overall	Degree of periodontitis				
	n	No	Mild	Moderate	Severe	Total Periodontitis
Total		121 (12.17%)	18 (1.81%)	575 (57.85%)	280 (28.17%)	873 (87.83%)
Gender						
Female	558 (56.1%)	83 (14.8%)*	14 (2.5%)	333 (59.6%)	128 (22.9%)*	475 (85.1%)*
Male	436 (43.8%)	38 (8.7%)	4 (0.9%)	242 (55.5%)	152 (34.8%)	398 (91.2%)
Age, years		39.1 ± 2.9^†^	37.5 ± 2.2^‡§^	39.8 ± 2.9^†^	40.3 ± 2.8^‡§^	
Smoking status						
Never/Former smokers	556 (55.9%)	82 (14.7%)*	13 (2.3%)	314 (56.4%)	147 (26.4%)	556 (85.2%)*
Current smokers	438 (44.0%)	39 (8.8%)	5 (1.1%)	261 (59.5%)	133 (30.3%)	438 (91.1%)
Education						
≤ 12 years	765 (76.9%)	88 (11.0%)	11 (1.4%)	443 (57.9%)	223 (29.1%)	470 (88.5%)
> 12 years	229 (23.0%)	33 (14.4%)	7 (3.0%)	132 (57.6%)	57 (24.8%)	196 (85.5%)
Monthly income						
< 286.000 CLP	619 (62.2%)	68 (10.9%)	10 (1.6%)	364 (58.8%)	177 (28.5%)	551 (89.0%)
≥ 286.000 CLP	375 (37.7%)	53 (14.1%)	8 (2.1%)	211 (56.2%)	103 (27.4%)	322 (85.8%)
Diabetes mellitus						
Yes	52 (5.2%)	4 (7.6%)	1 (1.9%)	26 (50.0%)	21 (40.3%)*	48 (92.3%)
No	942 (94.7%)	117 (12.4%)	17 (1.8%)	549 (58.2%)	259 (27.4%)	825 (87.5%)
Caries						
Yes	855 (86.0%)	91 (10.6%)*	15 (1.7%)	498 (58.2%)	251 (29.3%)*	764 (89.3%)*
No	139 (13.9%)	30 (21.5%)	3 (2.1%)	77 (55.4%)	29 (20.8%)	109 (78.4%)

Data are presented as numbers (percentages) or means ± standard deviation (SD)

*Significant differences in the following variables: gender, smoking status, diabetes and caries (Chi-square test *p < 0.05*)

^†‡§^ Significant differences in age (Kruskal-Wallis test *p < 0.004167)*

 A total of 87.8% had periodontitis, mostly moderate or severe periodontitis. Men had a higher prevalence of periodontitis than women (91.2% versus 85.1%, *p* < 0.05) and a higher prevalence of severe periodontitis (34.8% versus 22.9%, *p* < 0.05). Individuals with severe periodontitis were significantly older than individuals without periodontitis or with slight periodontitis (p < 0.05). Current smokers had a higher prevalence of periodontitis than non-smokers/former smokers (91.1% versus 85.2%, *p* < 0.05). In regards to education and monthly income there were no significant differences between the different degrees of periodontitis (*p* > 0.05). The prevalence of diabetes was 5.2% and those with diabetes presented a higher prevalence of severe periodontitis (40.3% versus 27.4%, p < 0.05) (Table 1). Table 2 compares the periodontal status between the caries free group and the group with caries. Caries lesions were significantly more prevalent in participants with moderate and severe periodontitis. Table 3 shows the results of ordinal regression analysis. In the univariate model, age (OR = 1.09; CI = 1.05–1.14), being male (OR = 1.84; CI = 1.43–2.36), being a smoker (OR = 1.37; CI = 1.07–1.76), having diabetes (OR = 1.74; CI = 1.01–3.00) having 3 or 4 caries (OR = 1.77 CI = 1.14–2.74) and 5 or more caries (OR = 2.48; CI = 1.67–3.67) were associated with the severity of periodontitis. These associations remained positive in the multivariate adjusted model which is depicted in Table 4: age (OR = 1.10; CI = 1.05–1.14), males (OR = 1.69; CI = 1.31–2.18), current smokers (OR = 1.30; CI = 1.01–1.67), diabetes (OR = 1.87; CI = 1.07–3.27) 3 or 4 caries (3–4 Caries OR = 1.74 CI = 1.12–2.29) and 5 or more caries (OR = 2.47; CI = 1.66–3.67).

**Table 2:** Comparison of study participants according to periodontal status [number (%)] between the caries free group and the group with caries (D ≠ 0)

Variable	Caries free	Caries	*p*
n Severity of periodontitis	139 (13.9)	855 (86.0)	
No	30 (21.5)	91 (10.6)	*0.001
Mild	3 (2.1)	15 (1.7)	0.730
Moderate	77 (55.4)	498 (58.2)	0.579
Severe	29 (20.8)	251 (29.3)	*0.042
Total periodontitis	109 (78.4)	764 (89.3)	*0.001

Data are presented as numbers (percentages)

*Chi-square test, *p* < 0.05

**Table 3:** Ordinal logistic regression analysis (unadjusted) according to the CDC/AAP case definition (Eke et al., 2012)

Variable	Categories	Periodontitis	
		Univariable analysis (unadjusted)	
		OR [CI]	*p*-value
Age (years)		1.09 [1.05; 1.14]	< 0.001*
Gender (reference = Female)	Male	1.84 [1.43;2.36]	< 0.01*
Smoking (reference = non smoker/former smoker)	Smoker	1.37 [1.07;1.76]	0.010*
Education (reference ≤12 years)	> 12 years	0.76 [0.57;1.02]	0.078
Household income (reference < 286.000 CLP)	≥ 286.000	0.87 [0.67;1.12]	0.280
Diabetes (reference = absence)	Presence	1.74 [1.01;3.00]	0.044*
Caries (reference = no caries)	1 caries	1.24 [0.77;2.0]	0.361
	2 caries	1.36 [0.84;2.20]	0.199
	3–4 caries	1.77 [1.14;2.74]	0.010*
	≥ 5 caries	2.48 [1.67;3.67]	< 0.001*

OR, Odds ratio

CI, Confidence Interval

CLP, Chilean Pesos

*p < 0.05

**Table 4:** Multiple ordinal logistic regression analysis according to the CDC/AAP case definition (Eke et al., 2012)

Variable	Categories	Periodontitis	
		Multivariable analysis (Adjusted)	
		OR [CI]	*p*-value
Age (years)		1.10 [1.05; 1.14]	< 0.001*
Gender (reference = Female)	Male	1.69 [1.31; 2.18]	< 0.001*
Smoking (reference = non smoker/Former smoker)	Current	1.30 [1.01;1.67]	0.039*
Education (reference ≤12 years)	> 12 years	0.82 [0.61;1.11]	0.213
Diabetes (reference = absence)	Presence	1.87 [1.07;3.27]	0.026*
Caries (reference = no caries)	1 caries	1.27 [0.78;2.04]	0.325
	2 caries	1.37 [0.86;2.26]	0.171
	3–4 caries	1.74 [1.12;2.29]	0.013*
	≥ 5 caries	2.47 [1.66;3.67]	< 0.001*

OR, Odds ratio

CI, Confidence Interval

*p < 0.05

## Discussion

To date epidemiological oral health studies in adults in South America are lacking. This is the first study to analyse the co-occurrence of dental caries and periodontitis in a representative sample of Chilean adults between 35 and 44 using the standard case definitions of the CDC–AAP. We found that individuals with caries showed a considerably higher prevalence of periodontitis than individuals without caries (89.3% versus 78.4%). In particular, individuals with caries suffered from severe periodontitis more frequently than subjects without caries (29.3% versus 20.8%).

The prevalence of caries (DT ≠ 0) was 85.9%. Previous records in Chile, such as the first National Health Survey in Chile (ENS) [20], reported a lower prevalence of 75.5% in subjects between 25 and 44 years of age. However, in the ENS study, besides the wider range of age, the examiners were nurses trained by professional dentists from the Ministry of Health. Consequently, there were methodological discrepancies between the studies [21]. Nevertheless, the prevalence of untreated dental caries in Chilean adults is high, especially when compared to European countries. For example, Great Britain [22] and Finland [23] reported a prevalence of dental caries of 31 and 26% respectively, in adults between 30 and 44.

In regards to periodontal disease, 87.8% of our population had periodontitis, distributed as 1.8%, 57.8% and 28.1% with mild, moderate and severe periodontitis, respectively. This prevalence is higher than in most epidemiological studies of individuals of similar ages, including the age group of 30–49. West Germany [24] reported an overall prevalence of 17.6% for severe periodontitis and 33.3% for moderate periodontitis. Additionally, in the German national survey [25], 17.4% of adults (35–44) had severe periodontitis and 53.5% moderate periodontitis. Meanwhile, in a similar age group (35–49) in the US a prevalence of severe and moderate periodontitis of 6.7% and 19.4% respectively, was reported [26]. Our findings confirm the high prevalence of periodontitis in Chilean adults compared to Europe and the US and provide a firm baseline for comparison with future studies to determine trends in periodontitis in Chilean adults.

As for the co-occurrence of dental caries and periodontitis, the question was whether there is a positive co-occurrence of dental caries and periodontitis in Chilean adults. We found a higher total prevalence of periodontitis as well as severe periodontitis in subjects with caries. Individuals with untreated caries presented a higher prevalence of severe periodontitis than those without caries (29.3% versus 20.8%). In other words, there was a relative change of approximately 40% in the prevalence of severe periodontitis among individuals with caries when compared to those without caries. To date, there is only one study from Finland to which we can compare our findings [10]. In that study, individuals with dental caries suffered severe periodontitis more frequently than those without caries (31% versus 16%) [10]. Most notably, and although the above-mentioned study used a different definition of periodontitis [10], the ratio of caries to periodontitis is similar in both countries. In order to obtain more insights into a possible link between caries and periodontitis, an ordinal regression analysis was performed. After the adjustment of confounding variables, the presence of 3 or 4 teeth with caries (OR 1.74) and the presence of 5 or more teeth with caries (OR 2.47) was positively associated with periodontitis, providing further support for our results. In line with these findings, the Finnish study also concluded that dental caries and particularly severe periodontitis occur in the same individuals [10]. In addition, age (OR 1.10), being male (OR 1.69), smoking (OR 1.01) and having diabetes (OR 1.87) were found to be positively associated with periodontitis in accordance with previous studies [27–29].

The co-ocurrence of both diseases might be explained by a series of adjustable risk factors associated with aspects of lifestyle and the accumulation of biofilm common for both caries and periodontitis. Our data nevertheless, showed no association between level of education, monthly income and the different degrees of periodontitis. Here, it has to be taken into account that theoretical models linking social determinants on oral health with causal pathways of the disease are missing [30]. Although our data cannot reflect the complexity of social processes, it is in accordance with the Commission on Social Determinants of Health by the World Organization of health who defined the most important stratifiers and proxy indicators as being income, education, occupation, gender and race/ethnicity. Indeed, the socioeconomic situation is a social determinant of health that influences health behaviours, such as smoking and access to healthcare [27–29]. In addition, low income is related to a higher risk of dental caries [6] and a higher prevalence of periodontitis [31]. In this context it should be mentioned that approximately 63% of Chilean adults have a monthly income of less than $286.000 CLP (approx. $380 USD), and among the Organization for Economic Co-operation and Development (OECD) member countries, Chile ranks the highest in income inequality. In addition, Chile shows the largest social inequality gradient in terms of tooth loss [32]. Moreover, and considering that there is no state subsidy for dental coverage, at least in this age range, access to the private health system is not affordable for most people. Therefore, it is reasonable that these upstream social determinants of health partly explain the co-occurrence of caries and periodontitis in the Chilenean population. In fact, It has been reported that social determinants of health have a greater impact than local factors [28] in terms of oral health. Surprisingly, Finland reported similar results regarding the co-occurrence of dental caries and periodontitis [10], even though it is one of the most equal countries regarding income distribution [33].

Even though the risk factors of caries and periodontitis are similar, there are differences in the microbiological profile. Bacterial species such as *Streptococcus mutans,* strongly associated with caries, produce acids that demineralize enamel and dentin [34]. These acids inhibit biofilm mineralisation and thus calculus formation which is normally produced in periodontitis. Unlike the cariogenic biofilm, the pathogenicity of the periodontopathogenic biofilm is related to its capacity to induce inflammation leading to periodontal breakdown [35, 36]. Hence, with regards to the microbiology and etiopathology more refined studies are needed to better understand the underlying factors of the positive co-occurrence of dental caries and periodontitis.

The major strengths of our study include the analysis of a national sample of Chilean adults between 35 and 44 with the novelty of assessing the co-occurrence of caries and periodontal disease. Additionally, the periodontal clinical parameters were reported using the standardised clinical case definition for population-based studies developed by the Centre for Disease Control and Prevention and the American Academy of Periodontology (CDC/AAP) [17, 18]. The present study provides a large dataset that will allow future comparisons, particularly for nationwide studies. Furthermore, the current findings might support the development of better strategies to tackle both caries and periodontitis thereby having an impact on oral health policies.

We recognize that this study has some limitations. First, owing to the cross-sectional nature of this study, a conclusion about the causal relation and the development of dental caries and periodontitis cannot be drawn. Prospective studies would likely overcome such shortcomings to some extent. Second, it remains unclear if this positive co-occurrence of both diseases changes with increasing age, since a relatively young cohort was analysed. Third, due to the high prevalence of caries in our population, we only used the D component of the DMFT index as the indicator of the prevalence of caries (DT ≠ 0) and not the percentage of DFMT. This might have influenced the present findings. Fourth, our findings represent only the Chilean population. With the exception of Finland, it remains unclear whether this co-occurrence pattern can be found in other populations. Fifth, the present study did not use the most recent classification of periodontal and peri-implant diseases [37]. However, this new classification has not yet been used for population-based surveillance of periodontitis thereby impeding the comparison with previous and future studies. Finally, the categorization of certain variables for the regression models including smoking habits could have also masked important differences. Similarly, auto-reported diabetes based on questionnaires can be variable and not accurate [38] therefore these findings should be interpreted with caution.

Given the lack of studies on the co-occurrence of caries and periodontitis, and considering that both diseases constitute a global public health problem [1, 2] future research should be performed in different populations with more age groups and, ideally, following a similar methodology, which would allow an appropriate comparison between populations. Furthermore, policy makers could assist in interpreting and act upon the findings, but this aspect is beyond the scope of the current report.

## Conclusion

Dental caries was positively associated with periodontitis in Chilean adults, especially in subjects with severe periodontitis. These data give an epidemiologic support for adopting new and better public health policies that would contribute to tackling both oral diseases.

## Data Availability

The datasets used and/or analysed during the current study are available from the corresponding authors on reasonable request.

## Abbreviations

CAL: Clinical attachment level
CDC–AAP: Center for Disease Control and Prevention and the American Academy of Periodontology
CI: Confidence interval
CLP: Chilean Pesos (CLP).
D: Decayed
DMFT: Decayed, Missing, Filled (DMFT) index
DT ≠ 0: Decayed different to zero
EFP: European Federation of Periodontology
ENS: National Health Survey in Chile
OECD: Organization for Economic Co-operation and Development
ORCA: European Organization for Caries Research
PD: Probing depth
SD: Standard deviation
SES: Socio-economic status
US: United States
WHO: World Health Organization
